# The reliability of the mini mental state examination and Montreal Cognitive Assessment for assessing cognitive impairment also depends on the type of cognitive deficits

**DOI:** 10.1590/1806-9282.20241310

**Published:** 2025-03-17

**Authors:** Josef Finsterer

**Affiliations:** 1 Neurology and Neurophysiology Center, Department of Neurology – Vienna, Austria.

Dear Editor,

We were interested to read the article by Silva et al. on a cross-sectional observational study comparing cognitive function assessed with the mini mental state examination (MMSE) and the Montreal Cognitive Assessment (MoCA) in 43 patients >60 years with heart failure^1^. Cognitive dysfunction was found with the MMSE in 26% of patients and with the MoCA in 23% of patients^1^. Family history of heart failure was positive in 54%, 30% were diabetic, and 84% had a history of arterial hypertension^1^. In total, 40% of patients had New York Heart Association (NYHA) II heart failure^1^. It was concluded that the MMSE performed better than the MoCA in detecting cognitive dysfunction in older patients with heart failure^1^. The study is impressive, but some points should be discussed.

First, it remains unclear why the conclusion was drawn that the MMSE performs better than the MoCA^1^. A higher percentage of abnormal findings on the MMSE does not necessarily mean that this test detects cognitive abnormalities better than the MoCA. Since both tests have their own focus in different cognitive areas (MoCA: short-term and working memory, retention, fluency, abstraction, executive functions, visual construction, attention, and orientation; MMSE: temporal and spatial orientation, memory and recall, attention, language and language comprehension, reading, writing, drawing, and arithmetic skills), it is conceivable that the included patients showed more deficits in areas that are predominantly detected with the MMSE than with the MoCA. Furthermore, both tests are used as screening methods, but an in-depth assessment of cognitive deficits requires the use of specific test batteries, such as the Cognitive Assessment Battery (CAB), the Brain Check Battery, the Addenbrooke's Cognitive Examination III (ACE-III), the Mini-Addenbrooke's (M-ACE), the Memory Impairment Screen (MIS), or the Rowland Universal Dementia Assessment Scale (RUDAS)^2^.

The second point is that patients with hearing impairment were excluded from the study, but patients with visual impairment were apparently not^1^. Since performing the MMSE and MoCA requires visual integrity, we should know why patients with visual impairment were not excluded from the study.

The third point is that a number of the included patients had risk factors for cognitive impairment other than heart failure^1^. For example, almost a third of the patients had diabetes^1^. Therefore, we should know how many of the included patients had well- or poorly controlled diabetes. What were the HbA1c levels in the patients with diabetes? How often was diabetic encephalopathy the cause of cognitive impairment?

The fourth point is that the cognitive state before the development of heart failure was not reported. Considering that the included patients were over 60 years of age, it is conceivable that at least some of them suffered from cognitive dysfunction unrelated to heart failure. In order to assess whether heart failure played a causal role, it would have been useful to include only patients who definitely had no cognitive deficits prior to the development of heart failure.

The fifth point is that NYHA stages were not correlated with MMSE and MoCA. Since a causal relationship between heart failure and cognitive dysfunction is suspected, it would be useful to correlate these parameters.

The sixth point is that test-to-test reliability was not assessed. To assess whether the test results are reliable and reproducible, it would have been important to retest the 43 patients after some time. It would also be useful to know whether the tests were performed by a single researcher or by different doctors. If different doctors examined the patients, we should know the interobserver variability between the investigators.

The seventh point is that heart failure may be due not only to coronary artery disease or arterial hypertension, but also to a number of other causes, including hereditary causes, such as hereditary hypertrophic, dilated, or restrictive cardiomyopathy^3^. Since more than 50% of the included patients had a positive family history of heart failure^1^, it is essential to screen these patients for hereditary cardiomyopathies.

In summary, this interesting study has limitations that put the results and their interpretation into perspective. Addressing these limitations could strengthen the conclusions and support the results of the study. The accuracy of the MoCA and the MMSE in assessing cognitive impairment also depends on the specific deficits of the patients studied.

## Data Availability

All data are available from the corresponding author.
